# Molecular docking of bioactive compounds extracted and purified from selected medicinal plant species against covid-19 proteins and in vitro evaluation

**DOI:** 10.1038/s41598-024-54470-6

**Published:** 2024-02-14

**Authors:** Ayesha Khanum, Yamin Bibi, Ilham Khan, Ghazala Mustafa, Kotb A. Attia, Arif Ahmed Mohammed, Seung Hwan Yang, Abdul Qayyum

**Affiliations:** 1 Department of Biology, PMAS-Arid Agriculture University Rawalpindi, Rawalpindi, 46300 Pakistan; 2 Department of Botany, Rawalpindi Women University, Rawalpindi, 46300 Pakistan; 3 Department of Plant Sciences, Quaid-I-Azam University, Islamabad, 45320 Pakistan; 4 Department of Biochemistry, College of Science, King Saud University, P.O. Box 2455, 11451 Riyadh, Saudi Arabia; 5 Department of Biotechnology, Chonnam National University, Yeosu, 59626 Republic of Korea; 6 Department of Agronomy, The University of Haripur, Haripur, 22620 Pakistan

**Keywords:** Anticancer, Antioxidant, Bioactive compounds, Medicinal plants, Biological techniques, Computational biology and bioinformatics, Molecular biology

## Abstract

Bioactive compounds are secondary metabolites of plants. They offer diverse pharmacological properties. *Peganum harmala* is reported to have pharmaceutical effects like insecticidal, antitumor, curing malaria, anti-spasmodic, vasorelaxant, antihistaminic effect. *Rosa brunonii* has medicinal importance in its flower and fruits effective against different diseases and juice of leaf is reported to be applied externally to cure wounds and cuts. *Dryopteris ramosa* aqueous leaf extract is used to treat stomach ulcers and stomachaches. Each of these three medicinal plants have been indicated to have anticancer, antiviral, antioxidant, cytotoxic and antifungal effects but efficacy of their bioactive compounds remained unexplored. Study was aimed to explore In-vitro and In-silico anticancer, antiviral, antioxidant, cytotoxic and antifungal effects of bioactive compounds of above three medicinal plants. DPPH and ABTS assay were applied for assessment of antioxidant properties of compounds. Antibacterial properties of compounds were checked by agar well diffusion method. Brine shrimp lethality assay was performed to check cytotoxic effect of compounds. Molecular docking was conducted to investigate the binding efficacy between isolated compounds and targeted proteins. The compound isomangiferrin and tiliroside presented strong antioxidant potential 78.32% (± 0.213) and 77.77% (± 0.211) respectively in DPPH assay while harmaline showed 80.71% (± 0.072) at 200 µg/mL in ABTS assay. The compound harmine, harmaline and PH-HM 17 exhibited highest zone of inhibition 22 mm, 23 mm, 22 mm respectively against *Xanthomonas* while Irriflophenone-3-C-β- D-glucopyranoside showed maximum zone of inhibition 34 mm against *E. coli.* The compound isomangiferrin and vasicine contained strong antibacterial activity 32 mm and 22 mm respectively against *S. aureus.* The compound mangiferrin, astragalin, tiliroside, quercitin-3-O-rhamnoside showed maximum inhibitory zone 32 mm, 26 mm, 24 mm and 22 mm respectively against *Klebsiella pneumoniae.* Highest cytotoxic effect was observed by compound tiliroside i.e. 95% with LD_50_ value 73.59 µg/mL. The compound tiliroside showed the best binding mode of interaction to all targeted proteins presenting maximum hydrophobic interactions and hydrogen bonds. The binding affinity of tiliroside was − 17.9, − 14.9, − 14.6, − 13.8, − 12.8 against different proteins 6VAR, 5C5S, IEA3, 2XV7 and 6LUS respectively. Bioactive compounds are significant natural antioxidants, which could help to prevent the progression of various diseases caused by free radicals. Based on molecular docking we have concluded that phytochemicals can have better anticancer and antiviral potential.

## Introduction

Bioactive compounds are secondary metabolites of plants. They offer diverse pharmacological properties due to great variety in their structures and play an important role in prevention and treatment of wide array of diseases from fever to dreadful cancers^1^. *Peganum harmala* L. is commonly known as Syrian rue and Harmal, belongs to family Zygophyllaceae^2^. *P. harmala* have pharmaceutical effects like insecticidal, antitumor, antimalarial, anti-spasmodic, vasorelaxant, antihistaminic effect. It shows different activities such as antioxidant, cytotoxic, antimutagenic, antifungal, herbicidal and fungicidal^3–5^. Plants also have anti-parasitic activity, anti-tumor, and hallucinogenic activities^6,7^. Fruit has been used for treatment of different diseases like asthma, jaundice, and colic^8^. Different bioactive compounds harmine, harmol, harmaline, and harmalol are beta-carboline alkaloids that are typically found in roots and seeds^9^. The isoxazole derivatives of harmine show anti-alzheimer activity^10^. Roots and seed extract of *P. harmala* were most biologically active, having strong anticancer and cytotoxic effects, presumably due to the presence of phytochemicals such flavonoids and phenolics^11^.

*Rosa brunonii* L*.* is a member of the Rosaceae family and is known as the 'Himalayan musk rose*'*, found in Himalayan region and Kashmir^12^. It is commonly known as ‘jungli gulap’ or ‘tarni’^13^. Flower and fruits of *R. brunonii* have medicinal importance as well as used as food^14^. Flower and fruits of this plant are effective against different diseases and digestive problems^15^. Crude extract from flower of *R. brunonii* show strong anti-oxidant potential. Leaf juice has been applied externally to cure wounds and cuts^12,16^. Different compounds extracted from fruit of *R. brunonii* like astragalin, quercetin-3-O-rhamnoside and tiliroside show strong anti-oxidant properties^15^. The phytochemicals of *R. brunonii* have cytotoxic effects^17^. The methanolic crude extracts of R. brunonii have cytotoxic effect and its fruits extract have antibacterial effect^18^.

*Dryopteris ramosa* is commonly known as Pakha, the member of family Dryopteridacea. It thrives in wet, shady environments. The fronds of *D. ramosa* are edible and can be taken orally for antibacterial and aphrodisiac effects^19^. The phytochemicals isolated from fronds of *Dryopteris ramosa have* strong anti-microbial effect^20^. Mangiferin and isomangiferin have been shown to exhibit antibacterial, immunomodulatory, anti-diabetic, anti-oxidative, anthelmintic, anti-inflammatory, anti-HIV, anticancer, anti-viral, anti-allergic, and macrophage-inducing actions^21^. The compound mangiferrin has hepatoprotective and analgesic abilities^22^. The isomangiferrin and mangiferrin have strong antioxidant potential^18^. Iriflophenone-3-C-β-d Glucopyranoside showed strong antibacterial potential against *E. coli K. pneumoniae, S. aureus* and low cytotoxic activity^23^. The present study aimed to investigate purified compounds for their antioxidant, antibacterial, cytotoxic and In silico analysis of purified compounds of medicinal plants. The study is novel in its type regarding combination studies of these compounds by in vitro and in silico assays for elucidating the pharmacological potential of native species of Pakistan against dreadful diseases caused by pathogenic bacteria as well as free radicals. This study is of particular importance in Pakistan, where the local population predominantly depends on these medicinal plants for the treatment of various diseases.

## Results and discussion

New therapeutic compounds can be synthesized using secondary metabolites found in medicinal plants. The types of secondary metabolites in solvent extract may vary depending on the solvent and extraction method. Different compounds like harmine, harmaline, vasicine, vasicinone (*P. harmala*) mangiferrin, isomangiferrin, irriflophenone -3-C-β-D D-glucopyranoside (*D. ramosa*) tiliroside, astragalin and quercitin-3-O rahmnoside (*R. brunonii*) were subjected for evaluation of their antioxidant, cytotoxic and antimicrobial potential. Molecular docking of these compounds was also done against different diseases (cancer, hepatitis B virus, influenza virus and SARS Covid 19).

**Figure 1:**
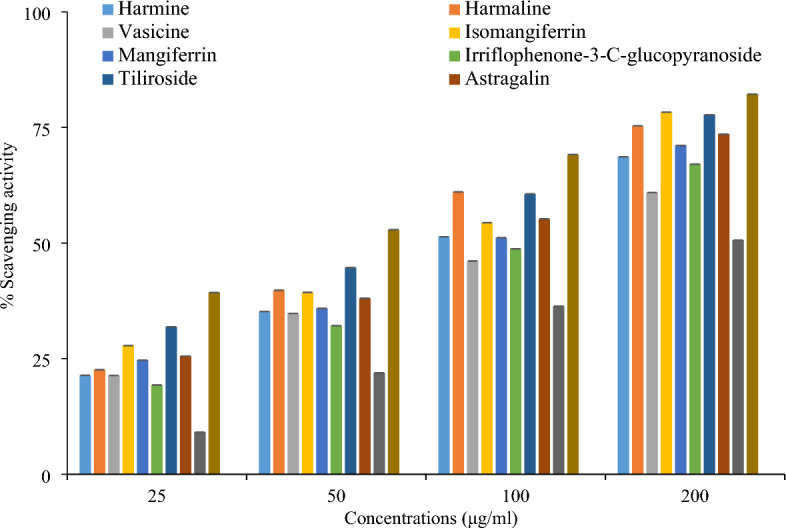
Comparison of antioxidant potential of bioactive compounds and ascorbic acid in DPPH assay.

DPPH and ABTS assays were used to check antioxidant potential. In DPPH assay all compounds showed significant free radical scavenging potential but the compound isomangiferrin and tiliroside exhibited highest antioxidant potential of 78.32% (± 0.213) and 77.77% (± 0.211) at 200 µg/mL (Fig. 1). In term of IC_50_ values isomangiferrin showed IC_50_ value of 93.85 µg/mL and tiliroside showed IC_50_ value 78.77 µg/mL that were less than ascorbic acid showed IC_50_ value 97.065 µg/mL. The low IC_50_ means strongest scavenging potential. It means that scavenging capacity of these compounds were strongest than ascorbic acid. Quercitin-3-O rhamnoside exhibited lowest 50% free radical scavenging potential with IC_50_ value 185.27 µg/mL concentration (Table 1). The IC_50_ value of compounds was calculated by linear regression equation (Table 1). The dark violet color changed to light yellow also predicted antioxidant potential of compounds. Our study is in correspondence to a previously conducted study in which isomangiferrin and tiliroside showed strongest antioxidant potential in DPPH assay as compared to other compounds^15,21,24,25^.

**Table 1:** Calculation of regression equation, regression co-efficient and IC_50_ values of bioactive compounds and ascorbic acid in DPPH assay.

Bioactive compounds	Regression equation	Regression Co-efficient	IC_50_ (µg/mL)
Harmine	y = 0.2563x + 20.137	R2 = 0.9435	116.5158018
PH-HM 17	y = 0.2397x + 20.055	R2 = 0.9336	124.9269921
Vasicine	y = 0.2098x + 21.119	R2 = 0.9344	137.6596759
Harmaline	y = 0.2833x + 23.132	R2 = 0.891	94.83939287
Isomangiferrin	y = 0.2798x + 23.739	R2 = 0.9858	93.85632595
Mangiferrin	y = 0.2566x + 21.667	R2 = 0.9741	110.4169914
Irriflophenone-3-C-β- D-glucopyranoside	y = 0.2616x + 17.288	R2 = 0.9547	125.0458716
Tiliroside	y = 0.2501x + 30.299	R2 = 0.9493	78.772491
Astragalin	y = 0.2638x + 23.376	R2 = 0.9554	100.9249431
Quercetin-3-O- rahmnoside	y = 0.2238x + 8.5356	R2 = 0.9308	185.2743521

**Figure 2:**
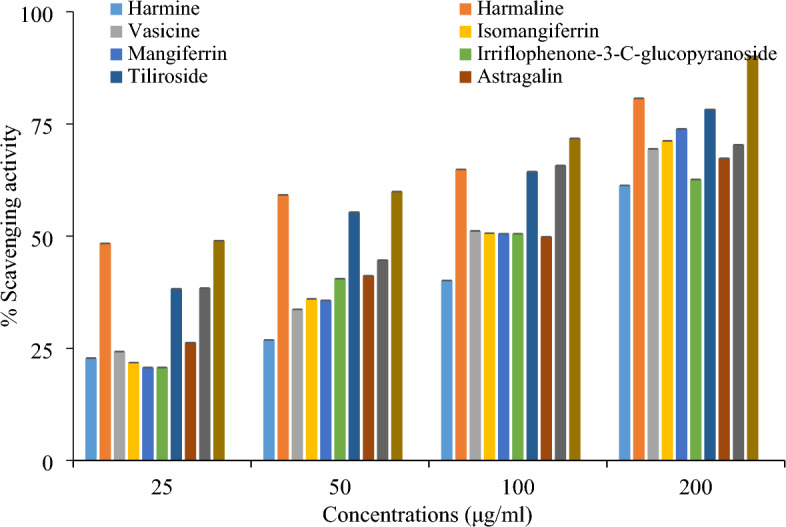
Comparison of antioxidant potential of different bioactive compounds of selected plant species and ascorbic acid in ABTS assay.

Another assay 2,2 -azino-bis-3-ethyibenzothiazoline-6-sulfonic acid was used to determine the free radical scavenging potential of compounds. All compounds showed significant free radical scavenging potential by ABTS assay. The maximum activity was shown by compound of *P. harmala* (harmaline) i.e. 80.71% with IC_50_ value 15.79 µg/mL. In terms of percentage and IC_50_ value others compounds (Astragalin, mangiferrin, Irriflophenone-3-C-β- D-glucopyranoside, tiliroside, vasicine, Quercetin-3-O- rahmnoside, isomangiferrin and harmine) showed significant free radical scavenging potential i.e. 78% (49.27 µg/mL), 73% (110.33 µg/mL), 71% (112.63 µg/mL), 70% (101.64 µg/mL), 69% (114.82 µg/mL), 67% (111.60 µg/mL), 62% (124.19 µg/mL) and 61.33% (148.26 µg/mL) respectively at 200 µg/mL (Fig. 2). More than 60% antioxidant activity was shown by all compounds. Ascorbic acid was used as standard and showed highest percentage (90%) with lowest IC_50_ value (14.77 µg/mL) as compared to all compounds (Fig. 2). The IC_50_ value of harmaline was near to IC_50_ value of ascorbic acid. The color was changed from bluish green to colorless that indicated the antioxidant activity of compounds. The antioxidant potential of all compounds and standard (Ascorbic acid) was expressed in terms of IC_50_ value. The linear regression equation was used to calculate IC_50_ values (Table 2).There was difference in antioxidant potential of compounds both in ABTS and DPPH assay may be due to presence to different radicals and solvent. The percentage scavenging activity increased with increase in concentration^26–28^.

**Table 2:** Calculation of regression equation, regression co-efficient and IC_50_ values of bioactive compounds and ascorbic acid in ABTS assay.

Chemical compound	Regression Equation	Regression Co-efficient	IC50 values
Harmine	y = 0.2239x + 16.804	R2 = 0.9974	148.2626172
Harmaline	y = 0.1706x + 47.306	R2 = 0.9597	15.79132474
Vasicine	y = 0.2536x + 20.88	R2 = 0.9686	114.8264984
HM 17	y = 0.2222x + 27.414	R2 = 0.8467	101.6471647
Mangiferrin	y = 0.2884x + 18.18	R2 = 0.9692	110.332871
Isomangiferrin	y = 0.21x + 23.919	R2 = 0.8402	124.1952381
Irriflophenone-3-C-β- D-glucopyranoside	y = 0.2676x + 19.859	R2 = 0.9621	112.6345291
Tiliroside	y = 0.1832x + 37.629	R2 = 0.8227	67.52729258
Astragalin	y = 0.204x + 39.948	R2 = 0.8869	49.40877598
Quercitin-3-O-rhamnoside	y = 0.2146x + 26.049	R2 = 0.9391	111.6076421
Ascorbic acid	y = 0.2241x + 46.689	R2 = 0.9738	14.77465417

**Figure 3:**
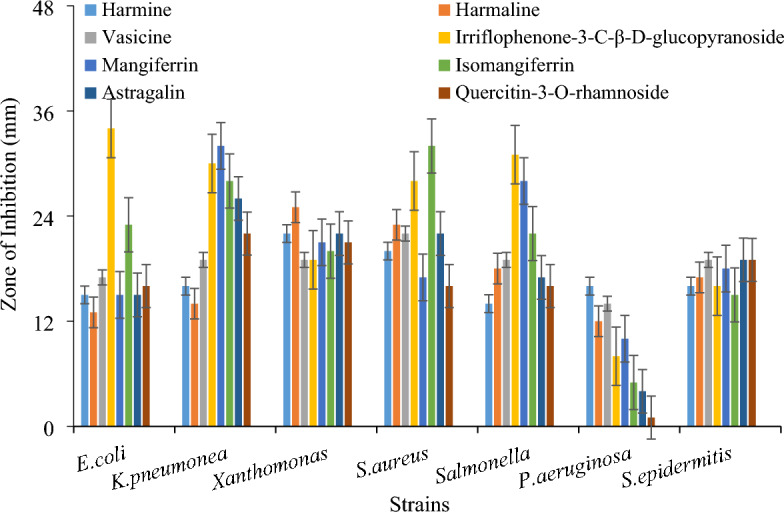
Comparison of antibacterial potential of bioactive compounds in agar well diffusion method.

Different compounds showed different inhibitory zones against different bacterial strains. Previously pharmacological actions of *P. harmala* including anti-oxidant, anti-tumor, hypoglycemic effect, leukemia healing, anti-inflammatory and analgesic properties, cytotoxic anti-tumor, and anti-microbial effects has been reported by Asgarpanah and Ramezanloo^29^. In present study the compound harmine and harmaline exhibited highest zone of inhibition 22 mm and 23 mm respectively against *Xanthomonas* while vasicine showed 22 mm against *S. aureus.* Irriflophenone-3-C-β- D-glucopyranoside showed maximum zone of inhibition 34 mm, 31 mm, 30 mm and 28 mm against *E. coli, Salmonella, K. pneumonia* and *S. aureus* respectively while less active against *P. aeruginosa, S. epidermitis* and *Xanthomonas* with inhibitory zones 8 mm,16 mm and 19 mm respectively. Mangiferrin was more active against K*. pneumonia* (32 mm) and *Salmonella* (28 mm) and weak against *P. aeruginosa* (10 mm)*, E. coli* (15 mm). The compound isomangiferrin contained strong antibacterial activity against *S. aureus* (32 mm) and *K. pneumonia* (28 mm) while lowest against 5 mm against *P. aeruginosa.* Astragalin, tiliroside and quercetin-3-O rhamnoside) showed strongest activity against *K. pneumonia* with inhibitory zone i.e. 26 mm, 24 mm, and 22 mm respectively. Similarly strongest antibacterial activity of Irriflophenone-3-C-β-D-glucopyranoside, mangiferrin and isomangiferrin was observed^21,23^. The compound of *R. brunonii* i.e. astragalin, tiliroside, quercitin-3-O-rhamnoside showed maximum inhibitory zone 26 mm, 24 mm, and 22 mm respectively against *Klebsiella pneumoniae*, while lowest against *P. aeruginosa.* Comparison of compounds antibacterial potential against different strains (Fig. 3). In previous study it has been observed that methanolic fruit's extract of *R. Brunonii* showed highest antibacterial potential against *Klebsiella pneumoniae* and lowest against *Bacillus subtilis*^18^.

**Figure 4:**
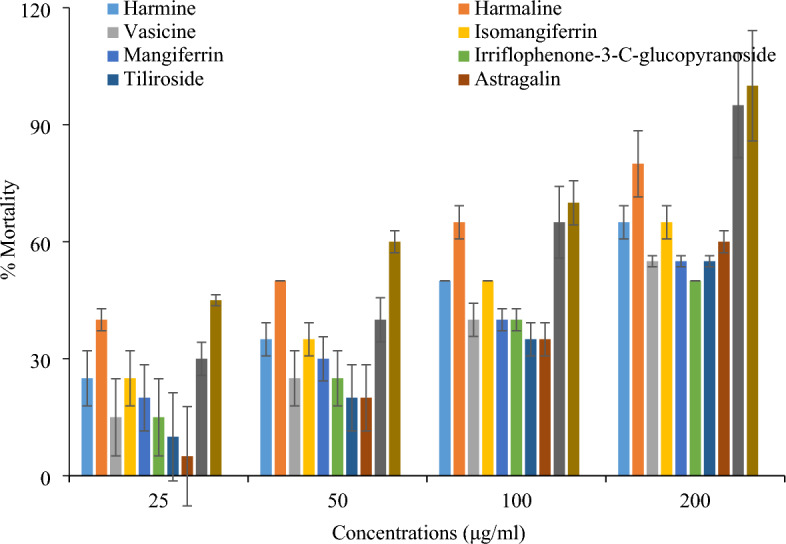
Comparison of cytotoxic potential of biological compounds in Brine shrimp lethality test.

For cytotoxic assessment of compounds brine shrimp lethality assay was used. The compound tiliroside exhibited 95% mortality rate with LD_50_ value 73.59 µg/mL highest mortality rate as compared to all other compounds. Harmaline showed 80% cytotoxic effect while harmine and Irriflophenone-3-C-β-D-glucopyranoside showed 65% mortality at highest 200 µg/mL concentration. The 55% was observed by vasicine, isomangiferrin and astragalin at 200 µg/mL. The lowest 50% cytotoxicity was showed by compound mangiferrin whereas 60% cytotoxic effect was observed by quercetin-3-O rhamnoside at 200 µg/mL (Fig. 4). Nicotine was used as standard. It showed 100 percent mortality rate at 200 µg/mL with lowest LD_50_ value 30.69 µg/mL among all compounds (Table 3). In previous literature cytotoxicity of mangiferrin and isomangiferrin has been observed^21^. Antibacterial, antioxidant and cytotoxic effects of some compounds were checked for the first time, but in previous literature same work has been done on whole plants and different fractions of these plants (*P. harmala, D. ramosa* and *R. brunonii*). These above different compounds were isolated from these plants. That’s why compounds also exhibited such potentials like antibacterial, antioxidant and cytotoxic potentials.

**Table 3:** Calculation of regression equation, regression co-efficient and LD_50_ values of bioactive compounds and ascorbic acid in BSL assay.

Chemical compound	Regression equation	Regression Co-efficient	LD_50_ values
Harmine	y = 0.2209x + 23.043	R2 = 0.9541	148.2626172
Harmaline	y = 0.1706x + 47.306	R2 = 0.9597	15.79132474
Vasicine	y = 0.2536x + 20.88	R2 = 0.9686	114.8264984
HM 17	y = 0.2222x + 27.414	R2 = 0.8467	101.6471647
Mangiferrin	y = 0.2884x + 18.18	R2 = 0.9692	110.332871
Isomangiferrin	y = 0.21x + 23.919	R2 = 0.8402	124.1952381
Irriflophenone-3-C-β- D-glucopyranoside	y = 0.2676x + 19.859	R2 = 0.9621	112.6345291
Tiliroside	y = 0.1832x + 37.629	R2 = 0.8227	67.52729258
Astragalin	y = 0.204x + 39.948	R2 = 0.8869	49.40877598
Quercitin-3-O-rhamnoside	y = 0.2146x + 26.049	R2 = 0.9391	111.6076421
Nicotine	y = 0.2241x + 46.689	R2 = 0.9738	14.77465417

In the process of drug discovery, computational innovations had a substantial impact. Computational biology emerged as an interdisciplinary approach integrating the computational techniques with biological systems to target different disease related challenges^30^.Virtual screening methods are frequently used to reduce the cost and time of medication development. Virtual screening predicts both binding modes and affinities of receptor and ligand based on docking and scoring of each molecule in a data set. Molecular docking has aided important drug discovery procedures. Docking approaches aid in the identification of correct ligand poses in a protein's binding pocket as well as the prediction of ligand–protein affinity. Finally, docking is a three-dimensional process for fitting two molecules together^31–35^.

In this study, molecular docking of different compounds that were previously reported from three medicinal plants was also done with target proteins (anticancer and antiviral). Similar study was reported by Gayathiri et al.,^30^ in *Sauropus androgynus* (L.) Merr against inflammation and cancer. The biochemical compounds were taken ligand to evaluate the antiviral and anticancer efficacy of these compounds. The 3D structures of antiviral and anticancer proteins were obtained from RCSB Protein Data Bank. Docking results were represented in the form of e-negative values (Tables 4 and 5). The arrangements of ligands (compounds) were based on ligand–protein binding energies. In molecular docking, higher e-negative values represent high binding affinity between the receptor and ligands molecules that indicated the higher efficacy of bioactive compound.

**Table 4:** Binding score of compounds against anticancer proteins 2XV7 and 5C5S.

Sr. No	Compound name	Binding energy (kcal/mol)2XV7	No of hydrogen bonds formed	Binding energy (kcal/mol)5C5S	No of hydrogen bonds formed
1	Tiliroside	− 13.8	4	− 14.9	2
2	Irriflophenone-3-C-β- D-Glucopyranoside	− 12.3	5	− 14.4	6
3	Mangiferrin	− 12.3	5	− 14.2	5
4	Quercitin-3-O-rhamnoside	− 11.5	2	− 13.2	5
5	Astragalin	− 11.3	1	− 14.3	4
6	Isomangiferrin	− 10.7	2	− 13.7	7
7	Harmine	− 7.3	2	− 8.3	1
8	Harmaline	− 7.3	2	− 8.3	1
9	Vasicine	− 7.0	2	− 7.5	No

**Table 5:** Summary of binding affinities of compounds against hepatitis B virus protein (6VAR), influenza virus protein (1EA3) and SARS covid-19 protein (6LUS).

Sr. No	Compound Name	Binding energy (kcal/mol) 6VAR	No of hydrogen bonds formed	Binding energy (kcal/mol) 1EA3	No of hydrogen bonds formed	Binding energy (Kcal/mol) 6LUS	No of hydrogen bond formed
1	Tiliroside	− 17.9	5	− 14.6	1	− 12.8	4
2	Mangiferrin	− 14.6	4	− 14.5	4	− 11.2	4
3	Quercitin-3-O-rhamnoside	− 14.4	4	− 13.8	5	− 12.6	4
4	Astragalin	− 14.3	3	− 12.2	4	− 12.2	2
5	Irriflophenone-3-C-β- D-Glucopyranoside	− 14.1	5	− 13.9	2	− 11.1	4
6	Isomangiferrin	− 14.1	7	− 12.4	4	− 11.6	5
7	Harmine	− 8.0	2	− 7.5	1	− 6.6	No
8	Harmaline	− 7.8	1	− 7.2	1	− 6.7	1
9	Vasicine	− 7.6	1	− 8.0	1	− 6.5	1

In current study the tiliroside compound that was extracted from plant *Rosa brunonii* showed best affinity towards all proteins (anti-cancer and anti-viral). In case of anticancer protein 5C5S tiliroside showed binding energies of − 14.9 kcal/mol and − 13.8 kcal/mol in 2XV7 anticancer protein (Table 4). Tiliroside showed binding affinities of − 14.6, − 17.9 and − 12.8 kcal/mol in case of influenza virus protein 1EA3, hepatitis virus protein 6VAR and SARS Covid- 19 protein 6LUS respectively (Table 5). The lowest binding affinity was shown by the compounds of *Peganum harmala*. Vasicine showed binding energies − 6.5, − 7.0, − 7.5, − 7.6 kcal/mol in case of SARS Covid- 19 protein 6LUS, anticancer protein (2XV7), anticancer protein (5C5S) and hepatitis virus protein (6VAR) respectively. In case of influenza virus protein (1EA3), compound harmaline showed lowest binding affinity of − 7.2 kcal/mol.

The sequence of compounds according to binding energy in case of anticancer protein (2XV7) was tiliroside > Irriflophenone-3-C-β- D-Glucopyranoside > mangiferrin > quercitin-3-O-rhamnoside > astragalin > isomangiferrin > harmine > harmaline > vasicine (Table 4). In case of second anticancer protein (5C5S) the sequence of compounds according to their binding energy was tiliroside > Irriflophenone-3-C-β- D-Glucopyranoside > astragalin > mangiferrin > isomangiferrin > quercitin-3-o-rhamnoside > harmine > harmaline > vasicine as shown in Table 4. The compounds sequence according to binding affinities in hepatitis protein (6VAR) case was Tiliroside > mangiferrin > quercitin-3-o-rhamnoside > astragalin > Irriflophenone-3-C-β- D-Glucopyranoside > Isomangiferin > harmine > harmaline > vasicine, while in case of influenza virus protein (1EA3) the sequence followed was Tiliroside > mangiferrin > Irriflophenone-3-C-β- D-Glucopyranoside > quercitin-3-o-rhamnoside > Isomangiferin > astragalin > vasicine > harmine > harmaline shown in Table 5.

For the first time, molecular docking of these compounds was carried out, targeting anticancer and antiviral proteins. This study is of particular importance in Pakistan, where the local population predominantly depends on these medicinal plants for the treatment of various diseases. Our research is a crucial step in drug development, especially in the context of SARS-CoV-2, a highly contagious disease prevalent today. Herbal medicines are not only cost-effective but also demonstrate long-term effectiveness with fewer side effects compared to chemically synthesized medicines. Additionally, previous studies have demonstrated the effectiveness of these plants in vitro in a variety of ways, including anticancer, antioxidant, antibacterial, and antiviral properties. The binding energies obtained by docking of 6LUS protein with ligands (compound) in a sequence (1) tiliroside (2) quercitin-3-o-rhamnoside (3) Astragalin (4) isomangiferrin (5) mangiferrin (6) Irriflophenone-3-C-β- D-Glucopyranoside (7) harmaline (8) harmine (9) vasicine was − 12, − 12.6, − 12.0, − 11.6, − 11.2, − 11.1, − 6.7, − 6.6, − 6.5 (Table 5). First time molecular docking of these compounds was done but in previous study, in vitro analysis of these plants on such activities like anticancer, antioxidant, antibacterial and antiviral has been exhibited^2,11,15,18,21,29,36–39^.

## Conclusion

Plant derived bioactive compounds have several therapeutic effects. Present study is highlighting the importance of bioactive compounds of *P. harmala, D. ramosa* and *R. brunonii* with significant antibacterial, cytotoxic and antioxidant potential in vitro and in silico that justified the ethnomedicinal importance of these plants. The compound tiliroside (*R. brunonii*) showed highest (95%) cytotoxic effect on brine nauplii among all compounds. The current study also explored the interaction of bioactive compounds to inhibit different cancer (2XV7 & 5C5S) and viral (Hepatitis 6VAR, SARS Covid 19 6LUS & Influenza IEA3) proteins. The results from this study displayed that compound tiliroside demonstrated the high binding affinity towards all cancer and viral proteins as compared to all other compounds. These bioactive compounds are significant sources of natural antioxidants, which could help to prevent the progression of various diseases, caused by free radicals i.e. certain cancers. In conclusion these results suggest that bioactive compounds may be helpful for the development of newer and more effective pharmaceutical components which may have lesser adverse effects.

## Material and methods

Isolated and purified compounds from different medicinal plants were used to test biological activities. The compounds were Harmine, Harmaline, vasicinone (PH 17) and Vasicine from *P. harmala,* Tiliroside, Astragalin, Quercetin-3-O-rhamnoside from *R. brunonii* and Mangiferin, Isomangiferin Irriflophenone-3-C-β-D-glucopyranoside from *D. ramosa.* These compounds were provided by Phytochemical laboratory of PMAS-Arid Agriculture University Rawalpindi, Pakistan.

### Antioxidant assessment

The antioxidant activities of compounds were analyzed by DPPH Free Radical Scavenging Assay and ABTS Assay.

#### DPPH (2–2-diphenyl-1-picrylhydrazyl) Free Radical Scavenging Assay

The DPPH assay was performed according to the protocol of Ghasemi et al.^40^ with little modifications. For DPPH solution, 3 mg of DPPH powder was dissolved in 100 mL methanol. 4 mg of compounds were dissolved in 5 mL methanol to obtained stock solution of compounds. Different serial dilutions (25, 50, 100, and 200 μg/mL) were prepared. The 3 mL DPPH methanolic solution was added to 400 μL compounds methanolic solution. The mixture was continuously shaken and stored at room temperature under shade for exactly 30 min. The absorbance of reaction mixture was measured at 515 nm spectrophotometrically. The dark violet color changed to light yellow color. The experiment was repeated three times and percentage inhibition was calculated as follows.

$${\text{Inhibition }}\left( \% \right) = \left( {{\text{Absorbance of control}}\, - \,{\text{Absorbance of sample}}} \right)/{\text{Absorbance of control}} \times {1}00.$$

IC_50_ value was calculated by linear regression equation that was obtained by plotting concentrations against percentage scavenging activity.

#### ABTS Assay (2,2 -azino-bis-3-ethyibenzothiazoline-6-sulfonic acid)

The ABTS test was performed using technique with some alternations^41^. The stock solutions (7 mM ABTS solution in 100 mL distilled water) and (2.4 mM potassium persulfate in 100 mL distilled water) were prepared. The ABTS solution was prepared by mixing equal parts of the two standard stock solutions and left them to react for 14 h at 25 °C in the dark. For compound solution, 4 mg of compounds were dissolved in 5 mL distilled water. Different concentrations (25, 50, 100 & 200 μg/mL) were prepared by using this stock solution. 400 μL compounds solution was allowed to react with 3 mL of the ABTS solution and the absorbance was taken after 30 min. Ascorbic acid was used as positive control. Color was changed from bluish green to colorless. The percentage scavenging potential was calculated by using following formula.

$$\left( \% \right){\text{ Inhibition}} = ({\text{Absorbance of control}} - {\text{Absorbance of sample}})/{\text{Absorbance of control }} \times { 1}00$$

Absorbance blank is absorbance of control (without compound) and Absorbance sample is absorbance of sample (compound).

IC_50_ value was calculated by linear regression equation that was obtained by plotting concentrations against percentage scavenging activity.

### Antibacterial activity

Antibacterial activity of compounds was determined by agar well diffusion method^42^. Antibacterial activity of different compounds was tested against seven clinical pathogens (*Klebsiella pneumonia, Escherichia coli, Xanthomonas, S. aureus, Salmonella, P. aeruginosa* and *S. epidermitis*). The nutrient agar medium was prepared by dissolving 6 g agar nutrient powder in 250 mL distilled water. The media was autoclaved and poured into pre-labeled petri plates under sterile condition. Then rinsed these plates with bacterial suspension before solidification. Allowed it to solidify for 15 min. Compound solution of 0.6 mg/mL was used. Cefixime with 1 mg/1 mL was used as positive control whereas DMSO was used as negative control. After solidification made wells of 6 mm using sterile cork borer. Each well was sealed with 20 μL of agar. After sealing 40 μL of each compound solution was added in well. The petri plates were sealed by para-film and kept in incubator for 24 h at 37 °C. The experiment was run in triplicates. The zone of inhibition of bacterial growth was measured with scale in mm.

### Cytotoxicity assessment

Brine shrimp lethality assay (BSLA) was used to check out the cytotoxic potential of different compounds of plant species by using the protocol described by Ishaque et al.^18^ with several modifications. Artificial sea water was prepared by dissolving 38 g sea salt in 1000 mL distilled water. This sea water was poured in a two-chamber container. One chamber was big and other was small. Added brine shrimp’s eggs on small side of chamber. Small side of chamber was covered and on other side provided illumination with electric lamp. Electric lamp was used to attract brine shrimp’s larva photo-tactically. Kept this for 24 h at room temperature for hatching of eggs. After 24 h brine shrimp’s larva matured to nauplii. Picked these nauplii from illuminated side with glass capillary tube for further process. The compounds were dissolved in Dimethyl sulfoxide (DMSO) and different concentrations of these compounds were prepared (25, 50, 100 and 200 μg/mL). Nicotine was used as positive control and same concentrations were made. Artificial sea water was used as a negative control. These concentrations were poured in vials and final volume of 5 mL was made by adding sea water. Added phototropic 20 nauplii in each vial with glass capillary tube. Then these vials were kept at room temperature for 24 h for incubation. After that alive nauplii were calculated. Mortality rate of brine shrimps was measured by using formula:

$$\% {\text{ of death}} = \left( {{\text{Total nauplii}}{-}{\text{Alive}} {\text{nauplii}} /{\text{Total nauplii}}} \right) \times {1}00$$

LC_50_ was calculated by drawing graph between percentage mortality and concentrations using excel.

### In silico analysis

Plant extracts possess thousands of bioactive chemicals, and determining the biological efficacy of each one in laboratory is too time consuming^43^. Therefore in silico evaluation of bioactive compounds from medicinal plants targeting cancerous and viral proteins has been done. Following steps were involved in *Insilico* analysis.

#### Molecular docking studies

The "key and lock" theory is utilized in molecular docking to discover the best match orientation for ligand and protein^44^. The structures of compounds were obtained from Pub Chem in SDF format. The phytochemicals show potential as drug candidates against the selected proteins by satisfying the Lipinski rule. Which entails a molecular weight below 500 g/mol, fewer than 10 H-bond acceptors, and fewer than 5 H-bond donors. Molecular weight, indicative of density, size, volume, and mass, is a key factor for therapeutic agents^45^. Furthermore, adherence to other rules, such as a molar refractivity between 40 and 130 and having fewer than 10 rotatable bonds, additional supports the drug-likeness^46^. Lipinski's rule-of-five, which connects physicochemical and pharmacokinetic indices, underlines that an orally active drug-like compound should not defy more than one of the subsequent criteria: hydrogen bond donors not exceeding 5, hydrogen bond acceptors not exceeding 10, molecular weight not exceeding 500 Da, and octanol–water partition coefficient (log P) not exceeding 5. Importantly, all selected phytochemicals adhere to these criteria. Anticancer (2XV7 and 5C5S) and antiviral proteins (hepatitis 6VAR, influenza 1EA3, SARS Covid-19 6LUS) were obtained from protein database. The target protein and ligands were synthesized according to normal ligand and protein preparation procedures, and the protein and ligand files were sent to Auto Dock vina. Each ligand's binding energy and binding contacts were determined. The docked complexes were studied using Ligplus.

#### Ligand preparation

The 3D structures of compounds (ligands) were obtained from Pub Chem compound database at NCBI in SDF format (Table 6). Then changed these SDF format structures to PDB format using pymol. Ligands were prepared by removing water molecules, adding hydrogen bonds, and required charges (Kollman and Gasteiger) using AutodockTools-1.5.7. Then saved structures of compounds in PDBQT format and used for docking by using AutodockTools-1.5.7^47^.

**Table 6:** Two dimensional structures of compounds with their Pub Chem CID and Molecular Weight.

Sr. no	Compounds	Formula	Molecular weight	Pub chem CID	Structures
1	Harmine	C13H12N2O	212.25	5,280,953	
2	Harmaline	C13H14N2O	214.26	3564	
3	Vasicine	C11H12N2O	188.23	667,496	
4	Mangiferrin	C19H18O11	422.3	5,281,647	
5	Isomangiferrin	C19H18O11	422.3	5,318,597	
6	Irriflophenone-3-C-β- D-glucopyranoside	C19H20O10	408.4	184,358	
7	Tiliroside	C30H26O13	594.5	5,320,686	
8	Astragalin	C21H20O11	448.4	5,282,102	
9	Quercitin-3-O rhamnoside	C21H20O11	448.4	5,353,915	

#### Protein preparation

**Figure 5:**
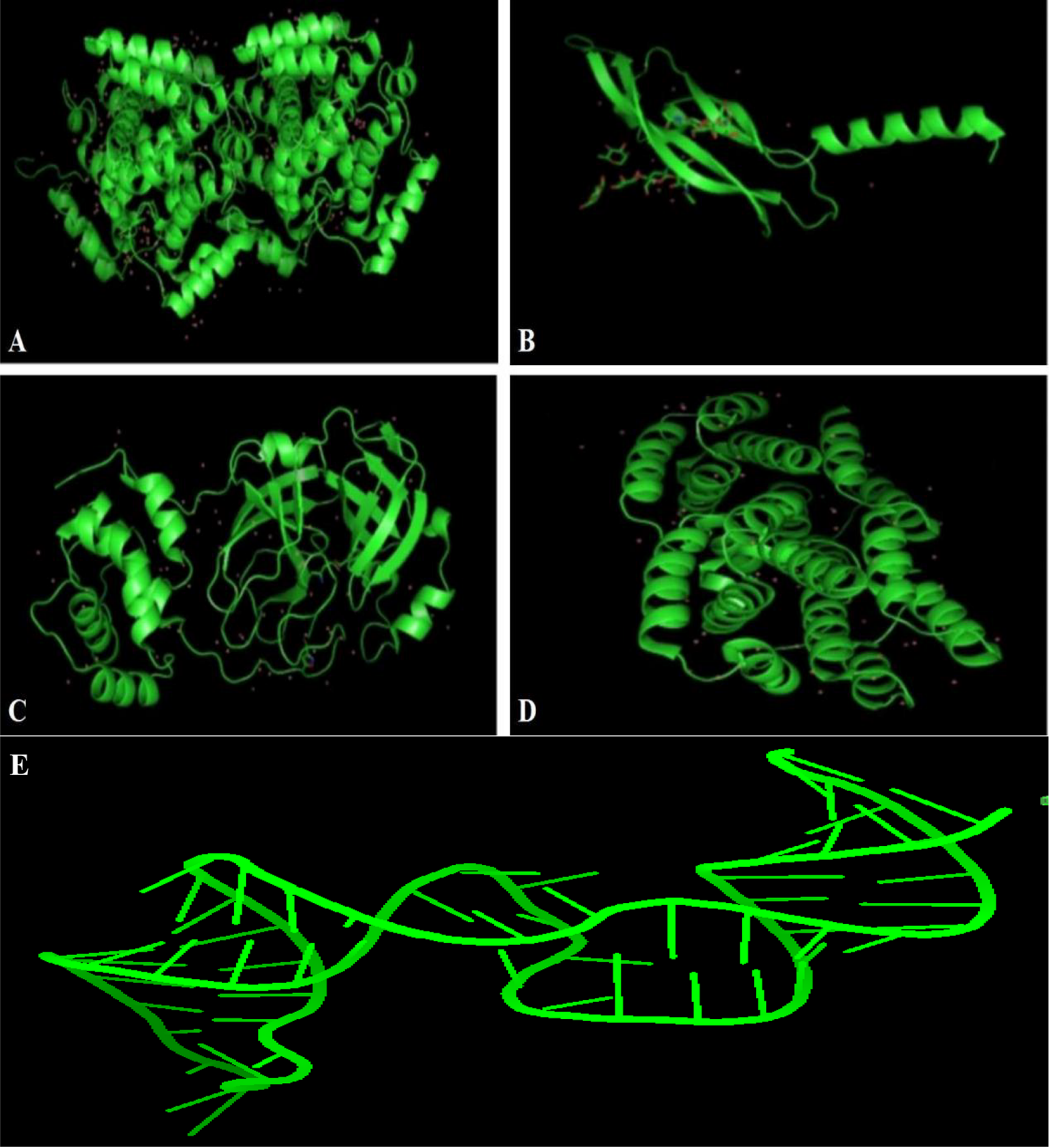
The 3D Structure of proteins (**A**) 5C5S anticancer (**B**) 2XV7 anticancer (**C**) 6LUS Covid- 19 Virus (**D**) 1EA3 influenza Virus (**E**) 6VAR hepatitis B Virus.

The crystal structures of different proteins were retrieved from RCSB Protein Data Bank in PDB format^48^. These proteins were anticancer (2XV7 & 5C5S) and antiviral (influenza 1EA3 & hepatitis 6VAR) (Fig. 5). These raw PDB proteins have only water molecules, metal ions, heavy atoms, and co-factors. These protein structures contained no information about formal atomic charges, bond orders and topologies so could not be used for molecular docking studies. So removed all water molecules, added polar hydrogen atoms and charges (Gasteiger and Kollman) in protein crystal structure using AutodockTools-1.5.7. Then optimized structure by assigning bond angles, bond orders and topology. Saved these structures in PDBQT format for further analysis.

#### The generation of grid box

Pre-calculated grid map is important in Auto Dock. Grid map helps in calculation of docking score. The docking evaluation results were analyzed by determining the amino acids in the active site. A three -dimensional grid box was created at maximum on proteins, to allow ligands to be docked on all areas of the receptor^49^. To generate grid box, selected both proteins receptor (5c5s.pdbqt, 6var.pdbqt, 2xv7.pdbqt, 1ea3 & 6lus.pdbqt) and saved ligands (compounds) in pdbqt format. Grid map was generated in such a way that both ligand and receptor fit into this three-dimensional matrix. Adjusted grid box size for X,Y,Z dimensions. The space was adjusted as 0.5 A that was nearly quarter of length for carbon single bonding atom. In the center of grid box ligand was present and was well-adjusted inside the active side of receptor proteins. Then exported the grid box dimension as grid.txt format. Then saved it in respective file for further processing.

#### Use of command prompt

Command Prompt is command line interpreter tool. The command is issued at the command prompt to examine the ligand-receptor binding affinity. The command prompt was open on desktop. Then provide command as “cd space the open folder of compound and add the link (E:\important\Molecular Docking\complete\cancer 2XV7) then enter “ and go to computer, open local disk C, open programme file, then open “The Scripps Research Institute” open vina, copy link and added this link (C:\Program Files\The Scripps Research Institute\Vina). After that insert \, click on double tab, space, –config, space, grid.txt, space, –log, space, log.txt and press the enter button. After that docking results of compounds were obtained after analyzing of bonding score.

#### The analysis of protein–ligand complex by Ligplus

**Figure 6:**
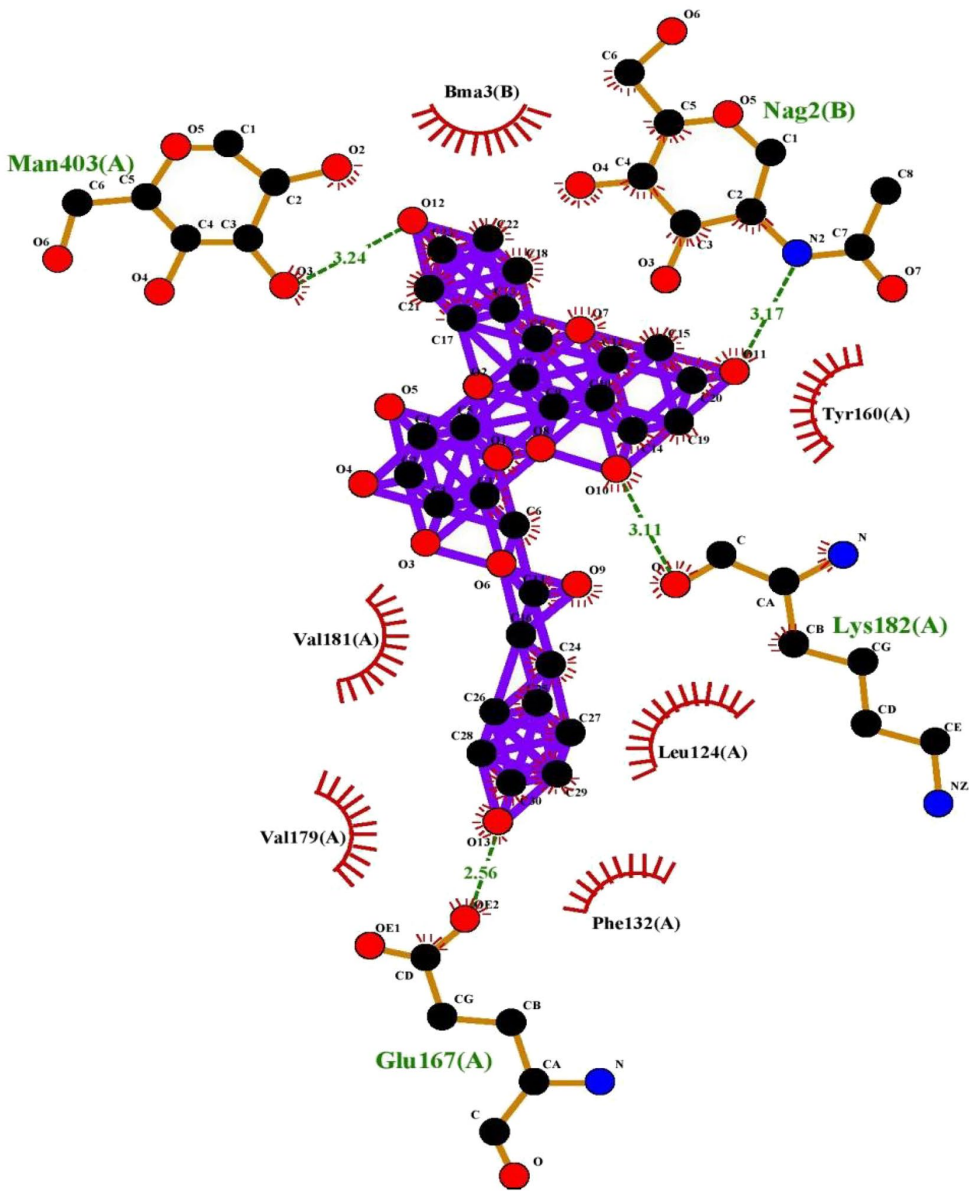
Interaction plot of tiliroside and 2XV7 cancer protein.

**Figure 7:**
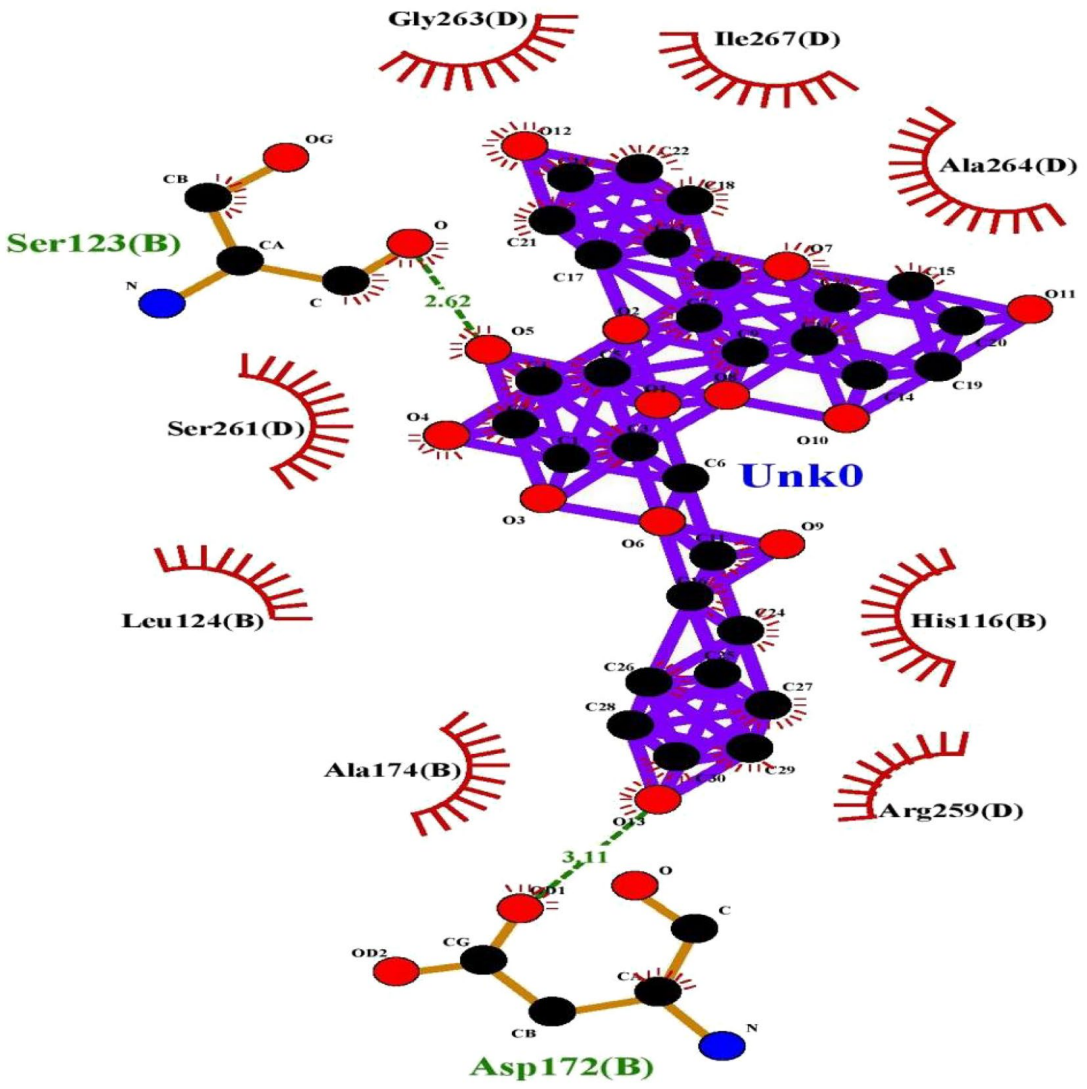
Interaction plot of Tiliroside and 5C5S cancer protein.

**Figure 8:**
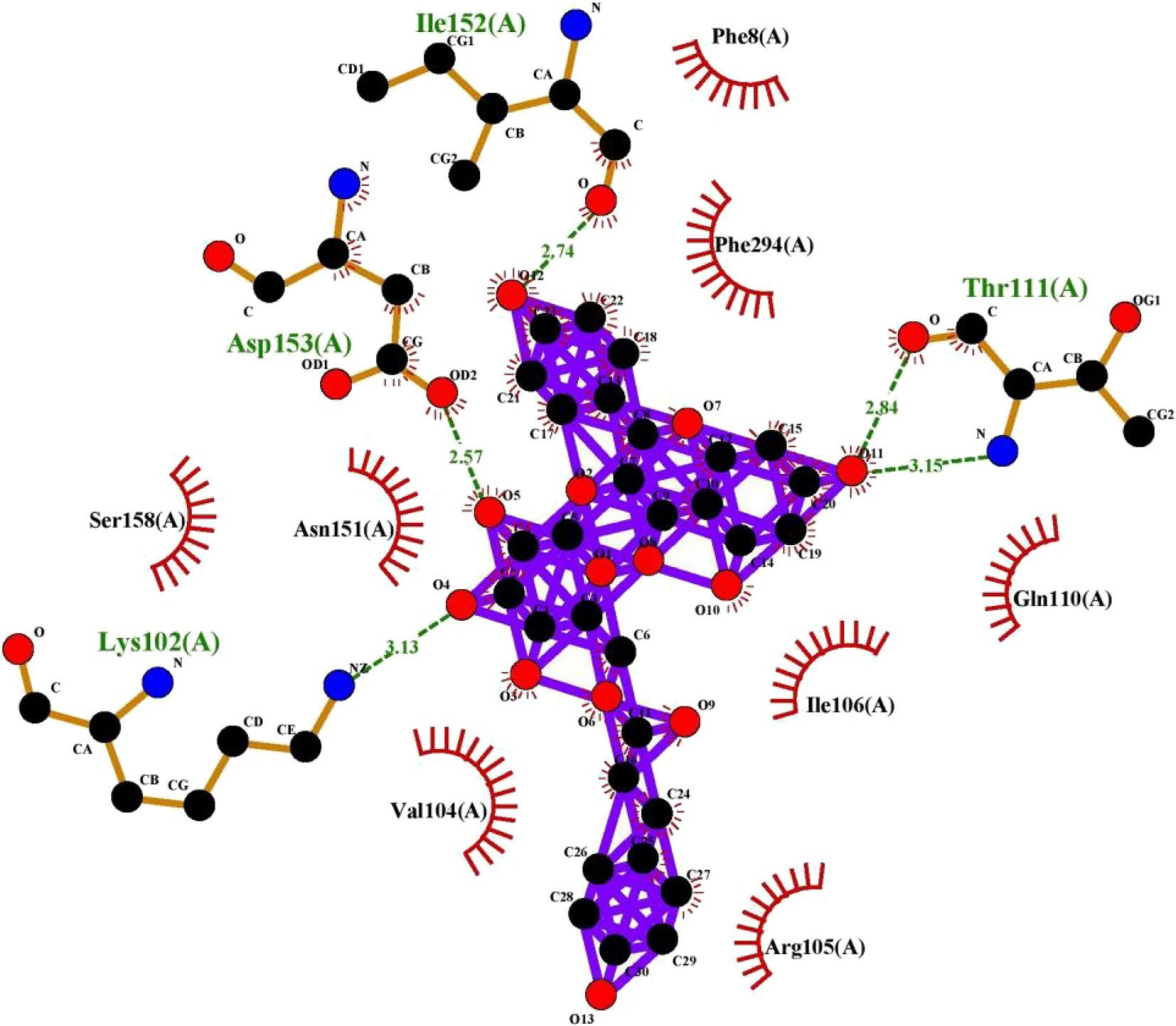
Interaction plot of compound Tiliroside and 6LUS protein (Covid-19 virus).

**Figure 9:**
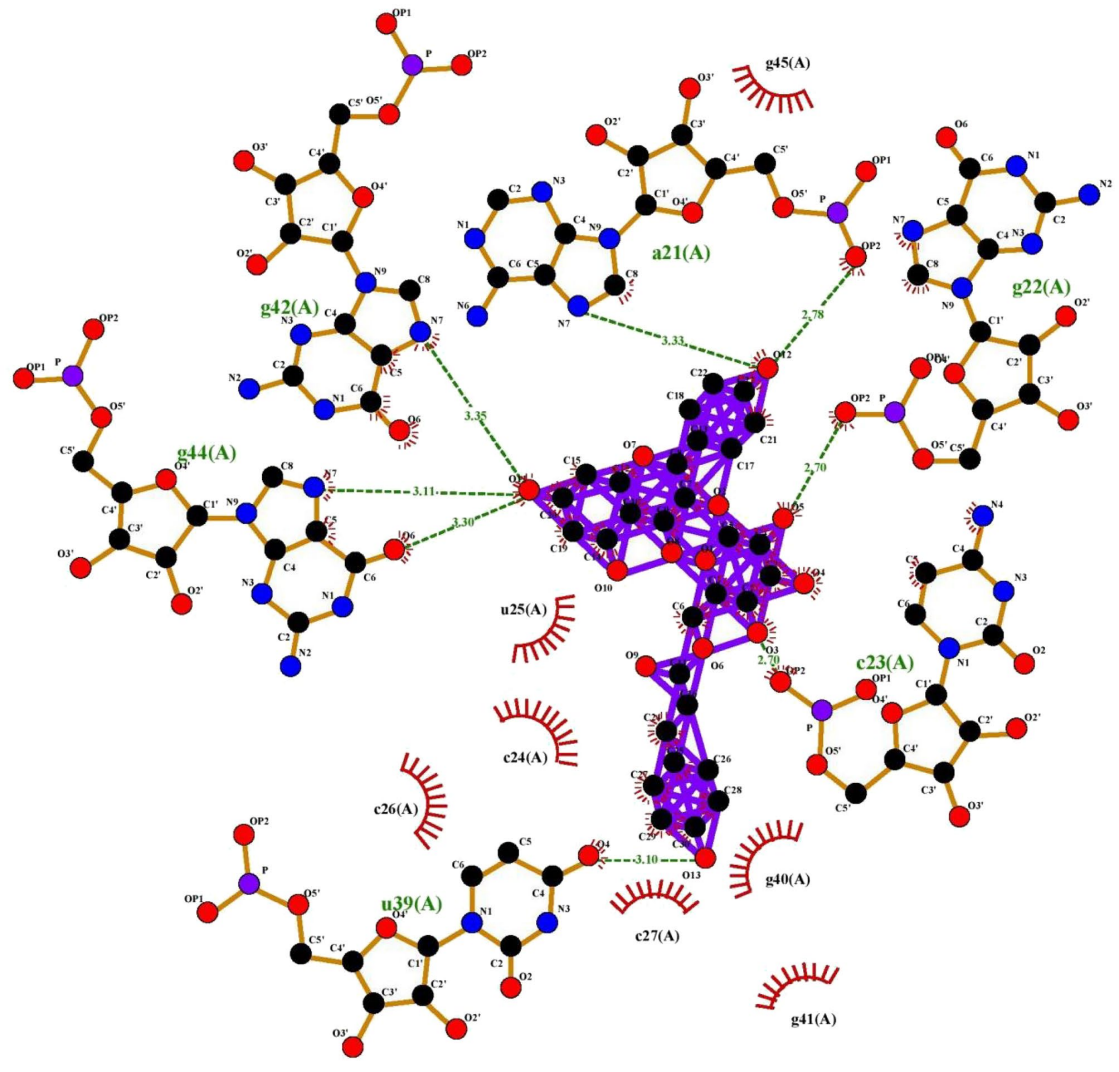
Interaction plot of ligand tiliroside and protein 6VAR (Hepatitis B virus).

**Figure 10:**
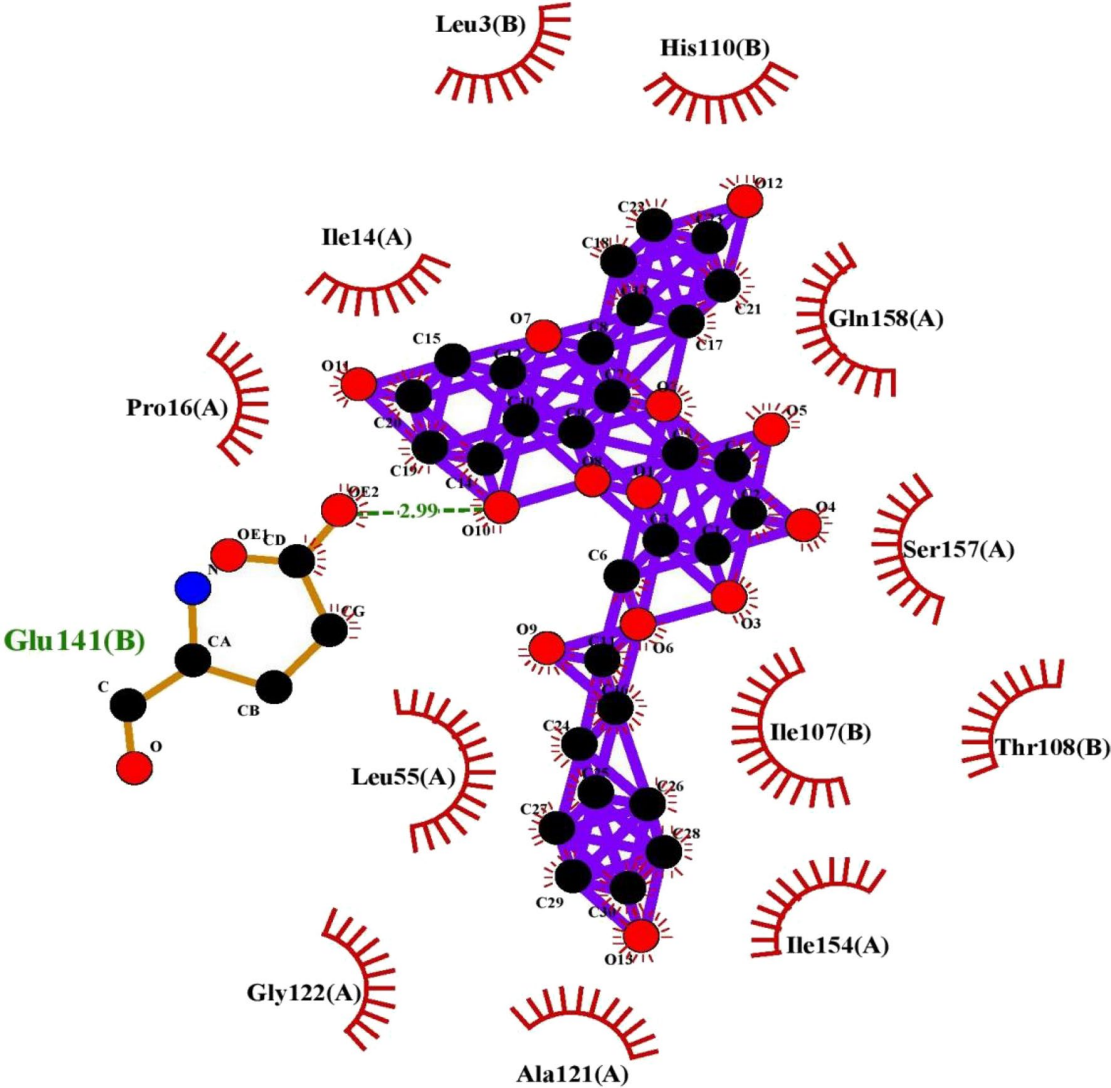
Interaction Plot of Tiliroside and receptor protein IEA3 (Influenza virus).

For assessing and characterization of interactions in our docked protein–ligand complex, LIGPLOT + version v.2.2.4 was used to visualize compounds with the best binding affinities. The hydrophobic and polar interactions between both the ligand and the target protein were visualized using this software (Figs. 6, 7, 8, 9, 10). The ligand.pdbqt and protein.pdbqt opened in pymol at same time. Then clicked on file and exported molecule, given name as complex, saved in pdb format in respective folder. Again clicked on file, exported image as PNG, saved and give any name. The image.oxps was converted online to jpg in respective folder. This method was used to show and generate interactions between compounds.

## Data Availability

The datasets used and/or analyzed during the current study are available from the corresponding author on reasonable request.

## Abbreviations

DPPH: 2-2-Diphenyl-1-picrylhydrazyl
ABTS: 2,2-Azino-bis-(3-ethylbenzothiazoline-6-sulphonic acid)
BSLA: Brine shrimp lethality assay
LD_50_: Median lethal dose.
IC_50_: Half maximal inhibitory concentration
(DMSO): Dimethyl sulfoxide
PM17: Vasicinone
